# Thermosensitive Micellar Hydrogels as Vehicles to Deliver Drugs With Different Wettability

**DOI:** 10.3389/fbioe.2020.00708

**Published:** 2020-07-17

**Authors:** Rossella Laurano, Monica Boffito

**Affiliations:** Department of Mechanical and Aerospace Engineering, Politecnico di Torino, Turin, Italy

**Keywords:** polyurethanes, hydrogels, smart vehicles, thermo-sensitivity, drug delivery systems, micellar gels, drug wettability, tunable release

## Abstract

The design of adaptable drug delivery systems able to encapsulate and release drugs with different wettability has been attracting widespread interest. Additionally, many attempts have been made to tune hydrophobic/hydrophilic drug release kinetics over time, avoiding the so-called *burst release*. In this scenario, hydrogels resulting from the assembly of micellar structures showing a hydrophobic core and a hydrophilic shell could represent a promising alternative to design versatile drug vehicles. In this regard, this work aimed at designing new thermosensitive micellar hydrogels starting from a custom-made amphiphilic poly(ether urethane) (PEU). Specifically, a commercial triblock copolymer (Poloxamer^®^ 407), selected to ensure the temperature-driven chain arrangement into micelles, was reacted with 1,6-diisocyanatohexane and 1,4-cyclohexanedimethanol. The successful PEU synthesis was proved by size-exclusion chromatography ($\bar{\text{M}}$_*w*_ 50000 Da) and infrared spectroscopy. Subsequently, the wettability-driven drug arrangement within the micelle network as well as the influence of drug-loading on the resultant formulation thermosensitivity was investigated by selecting ibuprofen (IBU) and ibuprofen sodium salt (IBUSS) as hydrophobic and hydrophilic drugs, respectively. Specifically, growing drug amounts were loaded into PEU solutions, and the average hydrodynamic micelle diameters and the critical micellar temperatures (CMT) were measured. Systems containing IBU at the highest tested concentration (i.e., 20 mg/mL) showed a significantly higher micelle average diameter (58.2 ± 4.7 nm) and a remarkably lower CMT (8.9°C) with respect to both the control (40.1 ± 1.4 nm and 21.6°C) and IBUSS-loaded formulations (37.3 ± 2.1 nm and 22.4°C). Then, the influence of drug encapsulation on the temperature at which micelles begin to aggregate was rheologically assessed, showing that IBU-loading induced a decrease in this parameter (14.6, 8.7, and 13.7°C for virgin, IBU-loaded, and IBUSS-loaded hydrogel, respectively). Finally, IBU and IBUSS releasing mechanism was analysed using the Korsmayer–Peppas model (*n* value of 0.63 ± 0.007 and 0.89 ± 0.003 for IBU- and IBUSS-loaded gels, respectively). Thanks to their micellar organisation, the here-developed hydrogel platform allowed the encapsulation of a high number of molecules with different wettability. Additionally, these systems exhibited tunable payload-releasing time without burst release and open the way toward the engineering of smart systems for the sustained co-delivery of multiple drugs in a target tissue/organ.

## Introduction

During the last years, the design of smart drug delivery systems has emerged as an ambitious challenge due to the need to (i) deliver drugs over time while reducing the administered dosage, (ii) reduce side effects associated to non-target tissues, and (iii) increase drug therapeutic efficacy in the pathological site. In this scenario, thermosensitive hydrogels are promising candidates as drug vehicles due to their ability to encapsulate therapeutic agents in the sol state, undergo a temperature-driven sol-to-gel transition entrapping drug molecules, and release their payload upon *in situ* injection (Gong et al., 2013; Dimatteo et al., 2018; Ghasemiyeh and Mohammadi-Samani, 2019). In addition, thermosensitive gels do not show cytotoxic effects associated with their gelation process, as they do not require neither chemical crosslinking agents nor potentially dangerous crosslinking reactions (e.g., photo-curing with UV light, in particular within the UVB and UVC range) to undergo gelation. However, two challenging issues are still attracting the attention of the research community working on this topic: (i) payload *burst release* upon hydrogel application and (ii) the difficulty in loading high amounts of hydrophobic drugs within hydrophilic networks (Hoare and Kohane, 2008; Larrañeta et al., 2018). With the aim to limit the *burst release* phenomenon, drug molecules have been bound to polymeric chains through covalent or non-covalent interactions. For instance, Andrade-Vivero et al. (2006) exploited the presence of the amino groups on 4-vinylpyridine or on *N*-(3-aminopropyl)methacrylamide monomers incorporated in poly(hydroxyethyl methacrylate) hydrogels to improve both the loading efficiency and the release kinetics of ibuprofen and diclofenac through electrostatic interactions occurring between the cationic functional groups of the polymer and the anionic drug molecules. In another work, Nuttelman et al. (2006) were able to tune dexamethasone release kinetics by covalently binding drug molecules to mono-acrylated poly(ethylene glycol) (PEG) chains, later incorporated into PEG-based hydrogels through photopolymerisation. Alternatively, the control over drug release can be carried out by tuning the crosslinking degree of interpenetrating polymer networks (IPNs) (Li et al., 2007), reducing the permeability of the external hydrogel surface (Matsusaki et al., 2007), or designing systems with an additional diffusive barrier for drugs (e.g., hydrogels containing drug-loaded particles). In this regard, Boffito et al. (2019b) recently reported a significant reduction of the initial drug burst release of approximately 85% through the design of hybrid sol-gel systems composed by an injectable thermosensitive hydrogel and ibuprofen-loaded mesoporous silica matrices. A similar approach was also reported by Chen et al. (2004), who designed injectable Poloxamer^®^-based hydrogels containing lidocaine-loaded microspheres. On the other hand, the approaches reported in literature to improve the loading efficiency of hydrophobic drugs usually consist of the introduction of hydrophobic domains within the hydrogel network or the addition to the gel solution of specific molecules (e.g., cyclodextrins) as drug *reservoir*. For example, Liu et al. (2007) reported an improvement in the loading efficiency and a reduction in diffusion-release rate of *p*-hydroxyanisole from hydrophobically modified poly(methacrylic acid) hydrogels. In another work, Zhang et al. (2005) were able to improve the release rate of hydrophobic drugs through their incorporation in acryolyl-β-cyclodextrins, later co-polymerised with *N*-isopropylacryl amide. However, in all the aforementioned studies the addition of specific polymeric segments to the hydrogel solution or further processing steps during hydrogel preparation were required to minimise drug burst release and/or to increase drug-loading efficiency.

In this scenario, the design of micellar hydrogels could simultaneously face both the previously discussed issues. Indeed, the presence of both hydrophilic and hydrophobic segments in the amphiphilic polymer chains allows the formation of micellar structures with a hydrophobic core and a hydrophilic shell upon temperature increase. As a consequence, this micellar organisation allows good interaction with both hydrophilic and hydrophobic drugs and a prolonged payload release over time. Among the different compositions of amphiphilic polymers reported in literature, poly(ethylene oxide)-poly(propylene oxide)-poly(ethylene oxide) (PEO-PPO-PEO) triblock copolymers are the most widely investigated for drug delivery applications (Bodratti and Alexandridis, 2018; Rey-Rico and Cucchiarini, 2018). These materials are FDA approved, commercially available (trademarks Poloxamer^®^ or Pluronic^®^) in a variety of different compositions (i.e., molecular weight and PEO content), and highly cytocompatible with no irritation after topical or parenteral administration. However, hydrogels based on Poloxamers^®^ usually suffer from poor residence time in aqueous environments (Boffito et al., 2016), negatively affecting drug release kinetics and leading to *burst release*. With the aim to overcome these drawbacks, in 2016 we reported the synthesis of a Poloxamer^®^ 407-based poly(ether urethane) which aqueous solutions exhibited faster gelation, higher mechanical performances, and improved stability in aqueous environment compared to thermosensitive hydrogels based on native Poloxamer^®^ 407 as such (Boffito et al., 2016). The capability of Poloxamer 407-based PEU gels to progressively release both hydrophilic and hydrophobic molecules as well as ionic species has been already reported (Boffito et al., 2019a, b). However, the literature lacks a thorough investigation of the interactions occurring between drugs and micellar structures. Thus, starting from the high promise of PEU-based hydrogels as drug delivery systems, in this work we characterised the interactions occurring between polyurethane micelles and drugs with different wettability at both the nano- and macro-scales. Furthermore, the effects of drug wettability on hydrogel thermosensitive behaviour and on drug release time were also studied. To this aim, a thermosensitive micellar hydrogel was designed starting from the synthesis of a custom-made amphiphilic poly(ether urethane) (PEU). Specifically, a poly(ethylene oxide)-poly(propylene oxide)-poly(ethylene oxide) triblock copolymer (Poloxamer 407) was selected as macrodiol to ensure polymer thermosensitivity, while 1,6-hexamethylene diisocyanate and 1,4-cyclohexanedimethanol were identified as diisocyanate and chain extender, respectively, to obtain a high molecular weight PEU (Pontremoli et al., 2018). Subsequently, drugs with different wettability (ibuprofen and ibuprofen sodium salt as hydrophobic and hydrophilic drug, respectively) were first loaded in PEU solutions at different concentrations. Then, their interactions with the temperature-induced micelle formation and nucleation processes was studied through the measurement of the average hydrodynamic micelle diameters and the estimation of the critical micellar temperature (CMT). In addition, both drug-loaded and not-loaded hydrogels were qualitatively and quantitatively characterised through tube inverting and gelation time tests and rheological analyses, respectively, to investigate the effect of drug encapsulation on hydrogel thermosensitivity and gelation process. Lastly, the drug release mechanism from gels embedding ibuprofen or ibuprofen sodium salt was assessed by means of drug release tests and the estimation of the release exponent values that characterise the drug transport mechanism according to the Korsmeyer–Peppas model.

## Materials and Methods

### Materials

Poloxamer 407 [P407, poly(ethylene oxide)-poly(propylene oxide)-poly(ethylene oxide) triblock copolymer, $\bar{\text{M}}{}_{n}$ 12600 Da, 70% w/w poly(ethylene oxide)], 1,4-cyclohexanedimethanol (CDM), 1,6-hexamethylene diisocyanate (HDI), and dibutyltin dilaurate were purchased from Sigma-Aldrich, Italy. Before use, reagents were treated according to the protocol proposed by Pontremoli et al. (2018) to remove residual water. Briefly, P407 was dried under reduced pressure (approximately 200 mbar) at 100°C for 8 h and then cooled down at room temperature under vacuum; HDI was distilled under reduced pressure; CDM was stored at room temperature (RT) in a desiccator; 1,2-dichloroethane (DCE) was poured over activated molecular sieves (3 Å, Sigma Aldrich, Italy) and maintained overnight under nitrogen atmosphere. All solvents were purchased from Carlo Erba Reagents (Italy) in analytical grade.

### Methods

#### Synthesis of Amphiphilic P407-Based Poly(Ether Urethane)

The P407-based poly(ether urethane) used in this work was synthesised following the pre-polymerisation method recently described by Laurano et al. (2019) (Supplementary Figure S1). Specifically, P407 was first dissolved in DCE (20% w/V concentration) at 80°C under continuous nitrogen flow. Then, HDI was added at 2:1 molar ratio with respect to P407 and the pre-polymerisation reaction proceeded for 150 min after the addition of a catalytic amount of dibutyltin dilaurate (0.1% w/w). Subsequently, the mixture was cooled down at 60°C and CDM (3% w/V in DCE) was added to the isocyanate-terminated prepolymer solution at 1:1 molar ratio with respect to P407. This second step of the reaction was carried on for 90 min and finally terminated through the addition of anhydrous methanol. The polymer was finally collected by precipitation in petroleum ether (4:1 volume ratio with respect to DCE total volume). To remove the catalyst and residual by-products, the polymer was then dissolved in DCE (20% w/V) and purified through precipitation in a mixture of diethyl ether/methanol (98/2 V/V, 5:1 volume ratio with respect to DCE). Finally, PEU was collected through centrifugation (Hettik, MIKRO 220R) at 0°C and 6,000 rpm for 20 min, dried overnight under the fume hood, and stored at 4°C under nitrogen atmosphere until use.

Hereafter, the synthesised PEU will be named CHP407, where C, H, and P407 identify the chain extender, the diisocyanate and the macrodiol, respectively.

#### Attenuated Total Reflectance Fourier Transform Infrared Spectroscopy

Attenuated Total Reflectance Fourier Transform Infrared (ATR-FTIR) spectroscopy was conducted on CHP407 samples to verify the success of the synthesis. Analyses were performed at RT using a Perkin Elmer spectrum 100 equipped with an ATR accessory (UATR KRSS) with diamond crystal. Spectra resulted from 32 scans in the range of 4,000 to 600 cm^–1^ with a resolution of 4 cm^–1^. Analyses were elaborated using the Perkin Elmer software and results are reported as average spectra of three different polymer batches.

#### Size Exclusion Chromatography

Size Exclusion Chromatography (SEC) analyses were performed through an Agilent Technologies 1200 Series (CA, United States) to estimate the molecular weight of the synthesised PEU. The instrument was equipped with a Refractive Index (RI) detector and two Waters Styragel columns (HR1 and HR4) conditioned at 55°C. *N,N*-dimethylformammide (DMF, CHROMASOLV Plus, inhibitor free, for HPLC, 99.9%, Carlo Erba Reagents, Italy), added with 0.1% w/V LiBr (Sigma Aldrich, Italy), was used as mobile phase. A calibration curve based on poly(ethylene glycol) standards was defined in the range of peak molecular weight M_*p*_ 4,000 – 200,000 Da. Before analyses, 2 mg of polymer were dissolved in 1 mL of mobile phase and filtered through a 0.45 μm syringe filter [poly(tetrafluoroethylene) membrane, Whatman]. Number Average Molecular Weight ($\bar{\text{M}}{}_{n}$), Weight Average Molecular Weight (${\bar{\text{M}}}_{w}$) and dispersity index (D) were estimated using the Agilent ChemStation software.

#### Dynamic Light Scattering

To thoroughly investigate the relationship occurring between drug loading and changes in micelle hydrodynamic diameter, dynamic light scattering (DLS) measurements were performed on CHP407 samples loaded or not-loaded with drugs characterised by a different wettability. Specifically, CHP407 was first dissolved at 0.5% w/V in physiological saline solution (0.9% NaCl); then Ibuprofen (IBU, Sigma Aldrich, Italy) or Ibuprofen Sodium Salt (IBUSS, Sigma Aldrich, Italy), as hydrophobic or hydrophilic drug, respectively, was added to PEU solution at 2, 5, 10, or 20 mg/mL concentration. Analyses were performed at 25, 30, and 37°C, according to the protocol recently published by Laurano et al. (2020), using a Zetasizer Nano S90 (Malvern Instruments, Worcestershire, United Kingdom) instrument. Before starting, samples were equilibrated at the test temperature for 15 min and then analysed according to Pradal et al. (2013). The reported hydrodynamic diameters resulted from the average of three different analysed samples. Data are reported as mean ± standard deviation.

Hereafter, drug loaded-CHP407 aqueous solutions (0.5% w/V) will be referred to as CHP407_IBU_Xmg/mL and CHP407_IBUSS_Xmg/mL, where X stands for the amount of encapsulated drug.

#### Critical Micellar Temperature

To investigate the effect of drug loading on the temperature at which micelle nucleation begins, the CMT of CHP407-based aqueous solutions was estimated using a fluorescent dye (1,6-diphenyl-1,3,5-hexatriene, DPH, Sigma Aldrich, Italy) as micellization marker. Samples (1 mL) were prepared by dissolving the polymer at 0.5% w/V concentration in physiological saline solution. Regarding the preparation of drug-loaded systems, IBU and IBUSS were first solubilised in ethanol and double-distilled water, respectively, at 40 mg/mL and then added to CHP407 solution (previously dissolved at slightly higher concentration) in order to reach the final volume of 1 mL and different drug concentrations (i.e., 2, 5, 10, and 20 mg/mL). Lastly, DPH (previously dissolved in methanol at 0.4 mM) was added to each sample at 10 μL/mL. Analyses were conducted according to the method described by Alexandridis et al. (1994). Briefly, virgin and drug-loaded solutions were heated between 5 and 40°C at 1°C/step, each step consisting of 5 min equilibration followed by UV/Vis spectra recording in the 500 nm to 300 nm spectral range (PerkinElmer, Lambda 25). Then, for each sample, the recorded absorbance values at 356 nm were plotted as a function of temperature obtaining a sigmoidal curve. The CMT was defined as the temperature corresponding to the first inflexion of this sigmoidal curve.

Hereafter, samples will be referred to as defined in the section “Dynamic Light Scattering.”

#### Preparation of Drug-Loaded Hydrogels

Virgin CHP407 hydrogels were prepared by dissolving the polymer at 15% w/V concentration in physiological saline solution overnight at 4°C according to the protocol published by Pontremoli et al. (2018). In order to prepare drug-loaded gels, an aliquot of IBU or IBUSS solution previously prepared in EtOH or double-distilled water respectively (40 mg/mL) was first added to physiological solution to reach a final drug concentration of 1 mg/mL and then, the resulting medium was used to solubilise CHP407 at 15% w/V concentration (Supplementary Figure S2).

Hereafter, IBU- and IBUSS-loaded gels will be referred to as CHP407_IBU and CHP407_IBUSS.

#### Tube Inverting Test

To qualitatively investigate the effect of drug encapsulation on hydrogel thermo-sensitivity, Tube Inverting Test was performed on all the formulated CHP407-based hydrogels. Specifically, samples were first prepared in bijou sample containers with an inner diameter of 17 mm (CarloErba reagents, Italy) as previously described (see paragraph “Preparation of drug-loaded hydrogels”); then, they were subjected to a controlled temperature increase from 4 to 40°C at 1 ± 0.1°C/step and 5 min equilibration time. At each step, samples were inverted to allow the visual inspection of the sol-to-gel transition. Sol and gel conditions were defined as “flow liquid sol” and “no flow solid gel,” respectively, within 30 s of observation. In order to better evaluate result variability due to operator’s perception of both sol and gel states, the test was performed by three different operators, each of them characterising a set of all formulated compositions. Results are reported as mean ± standard deviation.

#### Gelation Time at Physiological Temperature

Gelation time test at physiological temperature (i.e., 37°C) was performed to qualitatively evaluate the time required by loaded- and not-loaded hydrogels to undergo a sol-to-gel transition. Samples were prepared as previously described (see paragraph “Preparation of drug-loaded hydrogels”) and incubated at 37°C (Memmert IF75). At predefined time points (1–10 min, 1 min/step), hydrogels were inverted for 30 s to allow the visual inspection of the sol and gel states (defined as in the Tube Inverting Test). At the end of each step, samples were kept in an ice bath for 8 min to ensure that all the systems were in the sol state prior to the following incubation at 37°C. Result variability deriving from the qualitative evaluation of sol and gel conditions was investigated by asking to three different operators to characterise three different sets of formulations. Results are reported as mean ± standard deviation.

#### Rheological Characterisation

To thoroughly investigate hydrogel gelation mechanism and kinetics in the presence of hydrophobic or hydrophilic drugs, CHP407_IBU and CHP407_IBUSS were characterised using a stress-controlled rheometer (MCR302, Anton Paar Gmbh, Graz, Austria). The instrument was equipped with a 50 mm parallel plate geometry and a Peltier system for temperature control. CHP407 hydrogels were also analysed as control condition. Sol-to-gel transition was studied through temperature ramp tests carried out in the range 0°C–40°C, 2°C/min, 0.1 s^–1^ frequency. On the other hand, strain sweep tests were conducted at 37°C (10 Hz, strain range 0.01–500%) to investigate gel resistance to applied deformation. Lastly, frequency sweep tests were performed within the linear viscoelastic region (frequency range 0.1–100 rad/s, strain 0.1%) at 25, 30, and 37°C to characterise gel viscoelastic properties. For each analysis the sample was poured on the lower plate in the sol state at 0°C, heated at the test temperature and equilibrated for 10 min to reach thermal stability and finally tested.

#### Drug Release

The relationship between drug wettability and their release mechanism and timing from micellar hydrogels was studied through drug release tests conducted up to 14 days at 37°C. Specifically, drug-loaded hydrogels (1 mL) were prepared as previously described in paragraph “Preparation of Drug-Loaded Hydrogels” and incubated at 37°C for 15 min to ensure a complete sol-to-gel transition. Subsequently, 1 mL of Trizma^®^ (0.1 M, pH 7.4, Sigma Aldrich, Italy) previously equilibrated at the test temperature was added to each sample as release medium. Extracts were collected and completely refreshed at different time points (i.e., 15, 30, 45, 60, 90 min, 2, 3, 4, 5, 7 h, 1, 2, 5, 7, and 14 days). Released drugs were then quantified according to the method proposed by Alsirawan et al. (2013) using a High-Performance Liquid Chromatography (HPLC, Thermo Scientific, Dionex Ultimate 3000) instrument equipped with a C18 column (5 μm, 120 Å). Acetonitrile (ACN, CarloErba Reagents, Italy, HPLC grade) and phosphoric acid solution at 0.03% w/V were mixed at 60/40 V/V and used as mobile phase by setting 1.7 mL/min as flow rate. According to the selected mobile phase, 400 μL of each extract were added to 600 μL of ACN, to obtain a final Trizma^®^/ACN volume ratio of 40/60. Samples were then filtered through a 0.45 μm syringe filter [Macherey-Nagel, poly(tetrafluoro ethylene) membrane] and analyses were carried out at RT and 214 nm for 5 min with an injection volume of 20 μL. Subsequently, drugs were quantified by referring to a calibration curve based on IBU or IBUSS standards with concentration in the range of 0–1 mg/mL. Lastly, to better understand whether different release mechanisms occurred in the presence of IBU or IBUSS, the Korsmeyer–Peppas model was applied to the release data and the drug transport mechanism was defined based on the estimated release exponent *n* value (Dash et al., 2010).

#### Statistical Analysis

Statistical analysis was performed using GraphPad Prism 8.0 for MacOsX (GraphPad Software, La Jolla, CA, United States^1^). Two-way ANOVA analysis followed by Bonferroni’s multiple comparison test was used to compare results. The statistical significance of each comparison was assessed as reported in Boffito et al. (2016).

## Results

### Poly(Ether Urethane) Chemical Characterisation

ATR-FTIR spectroscopy performed on both the macrodiol and the synthesised polyurethane reported the appearance of new bands in CHP407 spectrum if compared to P407 one (Supplementary Figure S3). In detail, the peak at 1,720 cm^–1^ and 1,630 cm^–1^ can be ascribed to the stretching vibration of carbonyl groups; the band at 3,350 cm^–1^ can be attributed to the stretching vibration of N-H bonds, while at 1540 cm^–1^ the spectrum showed the bending vibration of N-H bonds together with the stretching vibration of C-N bonds. In addition, CHP407 showed an increased $\bar{\text{M}}{}_{n}$ compared to P407 (30,000 Da versus 10,000 Da) further confirming the success of the synthesis and a dispersity index of 1.6.

### Investigation of the Interactions Occurring Between PEU Micelles and Drugs With Different Wettability

#### Measurement of Micelle Average Hydrodynamic Diameter

**FIGURE 1:**
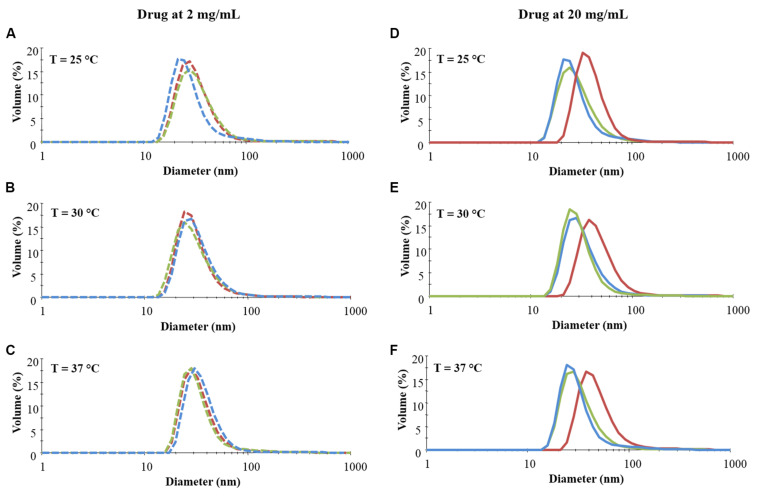
Distribution patterns (by volume) of micelle hydrodynamic diameter measured in CHP407 solutions at 0.5% w/V concentration not loaded- (blue line) or loaded with 2 (dashed line) and 20 (continuous line) mg/mL of IBU (red line) or IBUSS (green line). Analyses were performed at 25°C **(A,D)**, 30°C **(B,E),** and 37°C **(C,F)**.

To investigate the effect of drug loading on micelle nucleation and on their corresponding hydrodynamic diameter, DLS measurements were conducted at three different temperatures on samples loaded with different amounts of IBU or IBUSS. Virgin CHP407 solutions were also analysed as control condition. Specifically, Figure 1 reports DLS volume patterns of systems encapsulating the drugs at 2 and 20 mg/mL concentration, while results concerning CHP407 solutions loaded with IBU or IBUSS at 5 and 10 mg/mL concentration are illustrated in Supplementary Figure S4.

As reported in Figure 1 (left column), irrespective of the analysed temperature, no differences were observed between CHP407 and drug-encapsulating CHP407 profiles when systems were loaded at a low IBU or IBUSS concentration (i.e., 2 mg/mL). Upon an increase in drug content up to 5 mg/mL (see Supplementary Figure S4 – left column) an evident shift toward higher hydrodynamic diameter values was registered for samples containing IBU (red line) at 37°C, while no differences were observed at lower temperatures (i.e., 25 and 30°C) when compared to the control (blue line) and IBUSS-loaded CHP407 (green line) profiles. On the other hand, at the same payload concentration, IBUSS-loaded systems reported similar profiles with respect to the control at each analysed temperature. By further increasing drug concentration at 10 mg/mL and 20 mg/mL (see Supplementary Figure S4 and Figure 1 right columns, respectively), CHP407 solutions encapsulating IBU reported significant shifts toward higher hydrodynamic diameters at each tested temperature, thus suggesting that the introduction of a high amount of a hydrophobic drug favours the nucleation of bigger micelles. Conversely, no significant differences were observed between CHP407 and IBUSS-loaded CHP407 solutions irrespective of drug concentration and temperature.

**FIGURE 2:**
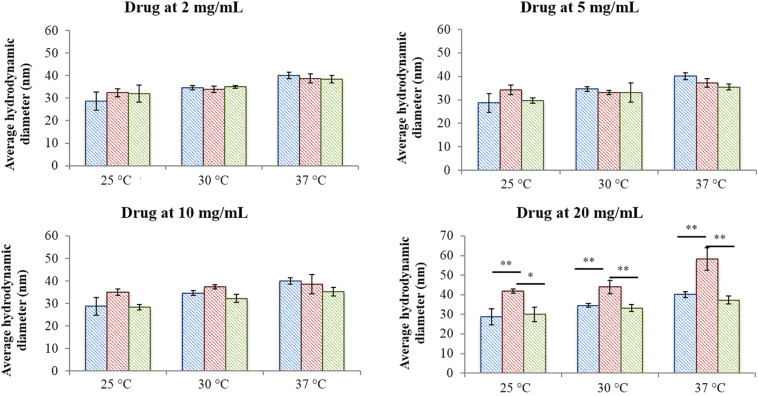
Average hydrodynamic diameters of micelles formed in CHP407 (blue), IBU- (red) and IBUSS-loaded (green) solutions (at 2, 5, 10, and 20 mg/mL drug concentration) at different temperatures (25, 30, and 37°C) (*p < 0.05, **p < 0.005).

Figure 2 reports micelle average hydrodynamic diameter measured at 25, 30, and 37°C upon an increase in drug content from 2 mg/mL up to 20 mg/mL. As expected, no differences were observed between the analysed systems when IBU and IBUSS were loaded at 2 mg/mL, with an average diameter around approximately 30, 35, and 40 nm at 25, 30, and 37°C, respectively. On the other hand, despite results observed in the volume patterns at 37°C for CHP407_IBU_5mg/mL (see Supplementary Figure S4C), micelles turned out to show similar average dimensions to those of CHP407 and CHP407_IBUSS_5mg/mL. Indeed, the average hydrodynamic diameters were measured to be 39.1 ± 1.4 nm, 37.2 ± 1.8 nm, and 35.4 ± 1.3 nm for CHP407, CHP407_IBU_5mg/mL, and CHP407_IBUSS_5mg/mL, respectively. Conversely, slight differences were observed at 25°C (35 ± 1.4 nm versus 28.7 ± 4 nm and 28.8 ± 1.2 nm for CHP407_IBU_5mg/mL, CHP407, and CHP407_IBUSS_5 mg/mL, respectively), thus suggesting that the addition of a hydrophobic drug favours the organisation of bigger structures at lower temperatures. Similar trends were also registered when drugs were encapsulated at 10 mg/mL, with an average hydrodynamic diameter slightly higher only for CHP407_IBU_10mg/mL at 25°C. Lastly, at the highest tested drug concentration (i.e., 20 mg/mL), the average hydrodynamic diameters of systems loaded with IBU turned out to be significantly higher compared to CHP407 and IBUSS-containing CHP407 solutions at each analysed temperature (e.g., average hydrodynamic diameters at 37°C were measured to be 40.1 ± 1.4 nm, 58.2 ± 4.7 nm, and 37.3 ± 2.1 nm for CHP407, CHP407_IBU_20mg/mL, and CHP407_IBUSS_20mg/mL, respectively).

#### Estimation of the Critical Micellar Temperature

**FIGURE 3:**
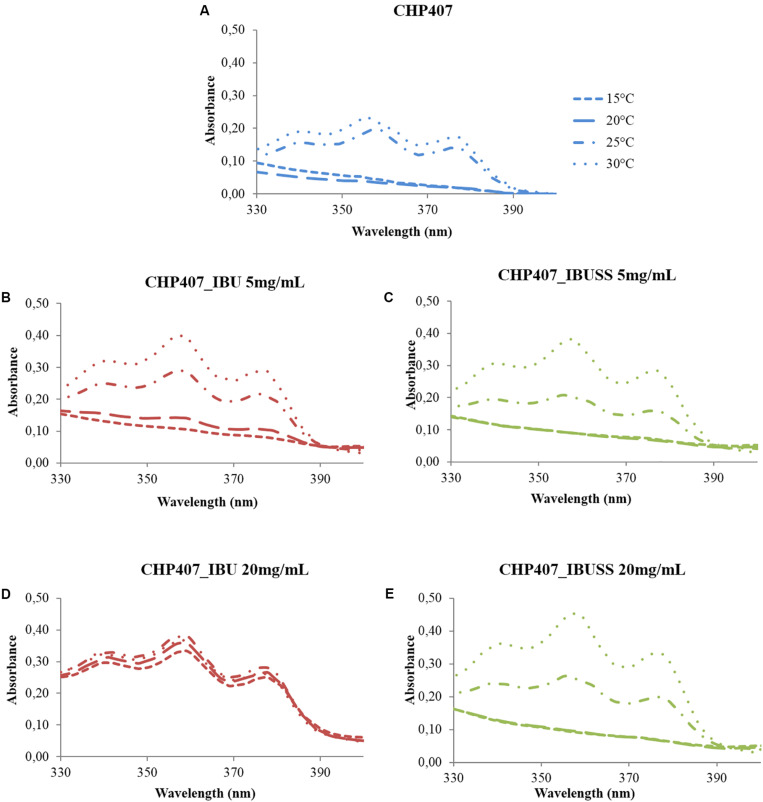
UV/Vis spectra of virgin CHP407 solution (0.5% w/V concentration) **(A)** and IBU- and IBUSS-loaded formulations (red and green line, respectively) at 5 mg/mL **(B,C)** and 20 mg/mL **(D,E)** upon temperature increase in the range 5–40°C.

The CMT was estimated for all considered formulations to investigate whether the presence of hydrophilic or hydrophobic drugs could affect the temperature at which micelles begin to nucleate. Figure 3 reports the UV/Vis spectra recorded for CHP407-based solutions at 0.5% w/V concentration loaded or not loaded with IBU or IBUSS at 5 mg/mL (Figures 3B,C) and 20 mg/mL (Figures 3D,E) recorded at different temperatures (i.e., 15, 20, 25, and 30°C). Data concerning systems loaded with drugs at 2 and 10 mg/mL concentration are reported in Supplementary Figure S5.

At low drug concentration, i.e., 2 mg/mL, no differences were observed between CHP407_IBU_2mg/mL and CHP407_IBUSS_2mg/mL as the temperature at which the intensity of the peak at 356 nm begins to increase turned out to be 20°C for both analysed systems (see Supplementary Figures S5B,C). In addition, this result was in accordance with that of CHP407 control solution, thus suggesting that the introduction of a low amount of drug did not affect micelle nucleation mechanism, irrespective of its wettability.

Upon an increase in drug loading at 5 mg/mL concentration (Figures 3B,C), slight differences appeared in IBU-loaded samples as the intensity of DPH signal at 356 nm turned out to increase at a lower temperature with respect to the control and CHP407_IBUSS_5mg/mL (i.e., 15°C versus 20°C). By further increasing drug concentration up to 10 mg/mL and 20 mg/mL, spectra of IBU-containing samples (see Supplementary Figure S5D and Figure 3D, respectively) were almost overlapped at each reported temperature. This evidence suggested that DPH solubilisation into the micelle core began at a very low temperature and that at 10°C its complete encapsulation was almost achieved. On the other hand, CHP407 samples loaded with IBUSS (see Supplementary Figure S5E and Figure 3E) showed similar trends to those reported for lower drug concentrations.

Lastly, CMT values were estimated for all considered systems (see Supplementary Figures S6, S7) according to Boffito et al. (2016) and reported in Table 1. As expected, similar CMT values were observed for CHP407-based solutions not loaded or loaded with 2 mg/mL of IBU or IBUSS. However, upon drug content increase at 5, 10, or 20 mg/mL, the presence of IBU sharply lowered the CMT value of the system (i.e., CMT = 8.9°C for CHP407_IBU_20mg/mL), thus indicating that hydrophobic molecules enhanced micelle nucleation at lower temperatures. Conversely, irrespective of drug concentration, the introduction of IBUSS did not affect the process, with respect to the control.

**TABLE 1:** Estimated critical micellar temperatures for virgin CHP407 solutions at 0.5% w/V concentration and drug-loaded formulations at 2, 5, 10, and 20 mg/mL concentration.

	Critical micellar temperature (°C)		
	CHP407 solutions	CHP407_IBU solutions	CHP407_ IBUSS solutions
0 mg/mL	21.6	–	–
2 mg/mL	–	21.8	22.7
5 mg/mL	–	19.2	22.5
10 mg/mL	–	10.7	22.6
20 mg/mL	–	8.9	22.4

### Effect of Payload Encapsulation on Hydrogel Thermosensitivity

#### Tube Inverting and Gelation Time Tests

**FIGURE 4:**
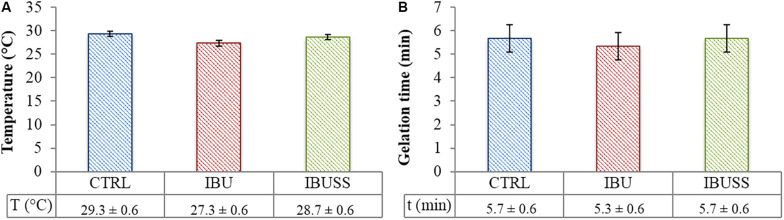
Results of Tube Inverting test **(A)** and Gelation Time test at physiological temperature **(B)** performed on CHP407 (blue), CHP407_IBU (red), and CHP407_IBUSS (green) hydrogels at 15% w/V concentration and loaded with 1 mg/mL concentration of drugs to evaluate their effect on the sol-to-gel transition process. Results are reported as mean ± standard deviation of three independent analyses performed by three different operators. Systematic errors of tube inverting test and gelation time test are ± 0.5°C and ± 30 s, respectively.

To qualitatively evaluate the influence of drug encapsulation on hydrogel thermo-sensitivity, tube inverting test and gelation time test at physiological temperature were performed on virgin hydrogels at 15% w/V concentration and on systems loaded with 1 mg/mL concentration of drug. Results are reported in Figure 4.

Regarding tube inverting test, drug loading seemed to slightly affect the gelation temperature of the hybrid systems irrespective of drug wettability. Indeed, gelation temperatures turned out to be 29.7 ± 0.6°C, 27.3 ± 0.6°C, and 28.0 ± 1°C for virgin CHP407, CHP407_IBU, and CHP407_IBUSS hydrogels, respectively. On the other hand, almost no differences were registered in their sol-to-gel transition time at 37°C.

#### Rheological Characterisation

**FIGURE 5:**
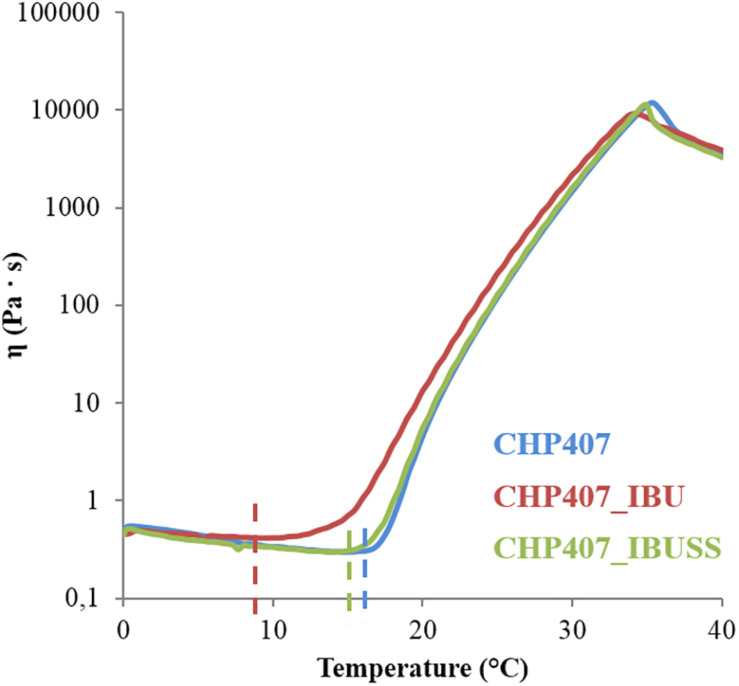
Trend of viscosity as a function of temperature within the range 0–40°C for virgin CHP407 (blue line) at 15% w/V concentration and for hybrid systems (CHP407_IBU and CHP407_IBUSS, red and green line, respectively) loaded with 1 mg/mL concentration of drug.

In order to thoroughly understand the effect of drug encapsulation on sol-to-gel transition upon temperature increase, a complete rheological characterisation was performed on virgin CHP407 hydrogels and on systems loaded with IBU or IBUSS. Figure 5 illustrates changes in solution viscosity recorded upon a controlled temperature ramp increase in the range 0–40°C.

In all analysed systems, viscosity initially decreased as a function of temperature according to the characteristic behaviour of sol systems subjected to a heating process. Then, a sharp viscosity increase was observed as a consequence of chain arrangement into an organised structure until complete sol-to-gel transition. In terms of trend of viscosity versus temperature, marked differences were observed among the investigated formulations, as the viscosity of CHP407_IBU (red line) started to increase at a lower temperature compared to the control and CHP407_IBUSS. Finally, the viscosity suddenly fell down at high temperatures (i.e., 35–36°C) due to gel melt fracture (Boffito et al., 2016), with no differences among the tested samples. The temperature at which the viscosity reaches the lowest value is defined as *T*_*onset*_. This temperature is a crucial gelation parameter as it marks the beginning of the micelle nucleation process. The *T*_*onset*_ values estimated for the analysed systems are reported in Table 2.

**TABLE 2:** *T*_*onset*_ and minimum value of measured viscosity (η_*min*_) estimated for virgin and drug-loaded systems prepared at 15% w/V concentration and loaded with 1 mg/mL concentration of drug.

	Gelation parameters	
	η _*min*_ (Pa⋅s)	*T*_*onset*_ (°C)
CHP407	0.3	14.6
CHP407_IBU	0.42	8.7
CHP407_IBUSS	0.3	13.7

The addition of IBUSS to CHP407 solutions did not significantly affect either the system minimum viscosity value or the *T*_*onset*_, which was measured to be 14.6 and 13.7°C for CHP407 and CHP407_IBUSS, respectively. Concerning CHP407_IBU, consistent differences were registered with respect to the control in *T*_*onset*_ value, thus suggesting that the introduction of hydrophobic molecules could affect gelation mechanism.

Strain sweep tests conducted at 37°C (Supplementary Figure S8) measured storage and loss moduli (G’ and G”, respectively) values as a function of applied deformation. While similar storage and loss moduli values (approximately 7 and 1.6 kPa for G’ and G”, respectively) were registered within the linear viscoelastic region (i.e., strain range in which G’ and G” are constant) for both unloaded- and drug-loaded gels, a significantly lower strain resistance was observed for CHP407_IBU (11.6% versus 18.6% strain at break for IBU-loaded and virgin/IBUSS-loaded gels, respectively), thus suggesting a different micelle organisation within the network in the presence of IBU.

**FIGURE 6:**
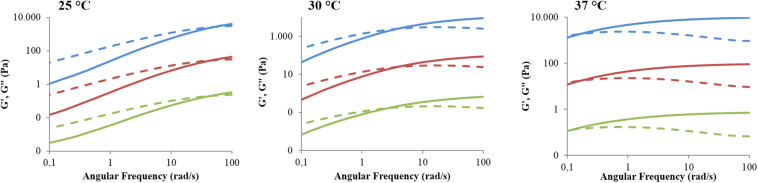
Trends of storage and loss moduli versus angular frequency within the range 0.1–100 rad/s for CHP407 (blue), CHP407_IBU (red), and CHP407_IBUSS (green) hydrogels at 25, 30, and 37°C. The storage (G’) and the loss moduli (G”) are reported as continuous and dashed lines, respectively. For high graph clarity, G’ and G” were shifted along the *Y* axis with respect to the control by a factor of 100 and 10,000 for CHP407_IBU and CHP407_IBUSS, respectively.

Lastly, frequency sweep tests at 25, 30, and 37°C allowed the investigation of hydrogel gelation kinetics through the variation of G’ and G” upon temperature increase (Figure 6). In general, G’ values lower than G” ones characterise a system in the sol phase; conversely, G’ values higher than G” ones are indicative of a gel behaviour. The frequency at which G’ becomes higher than G”, i.e., the crossover frequency (ω_*G*__’__/G__”__*crossover*_), marks the transition from the typical behaviour of a sol toward that of a gel. Those samples that exhibit a G’/G” crossover within the investigated frequency range are defined as biphasic systems. The analysed systems turned out to be almost completely in the sol state at 25°C, in a biphasic phase at 30°C, and almost completely in the gel state at 37°C, although a complete gel development was not achieved because G’ and G” were not frequency-independent. However, the crossover frequency values reported in Table 3 suggested different gelation kinetics, with respect to the control sample, in the presence of both hydrophobic and hydrophilic drugs. Indeed, at 25°C the crossover frequencies of CHP407_IBU and CHP407_IBUSS hydrogels (i.e., 50 rad/s and 45 rad/s, respectively) were measured to be at lower frequencies if compared to CHP407 gels (i.e., 58 rad/s). Upon temperature increase up to 30°C, CHP407_IBU and CHP407 reached the G’/G” crossover at the same angular frequency (i.e., 4 rad/s), while CHP407_IBUSS exhibited the G’/G” crossover at 2.5 rad/s. Lastly, at 37°C all systems showed almost the same ω_*G*__’__/G__”__*crossover*_, suggesting that all the formulations achieved the same degree of network organisation.

**TABLE 3:** Crossover frequencies between storage and loss moduli (ω_*G*__’__/G__”__*crossover*_) measured at 25, 30, and 37°C for virgin and drug-loaded CHP407-based hydrogels (drug concentration 1 mg/mL).

	ω _*G*__’__/G__”__*crossover*_ (rad/s)		
	25°C	30°C	37°C
CHP407	58	4	0.15
CHP407_IBU	50	4	0.13
CHP407_IBUSS	45	2.5	0.12

### Investigation of Payload Releasing Mechanism and Kinetics From Micellar Hydrogels

**FIGURE 7:**
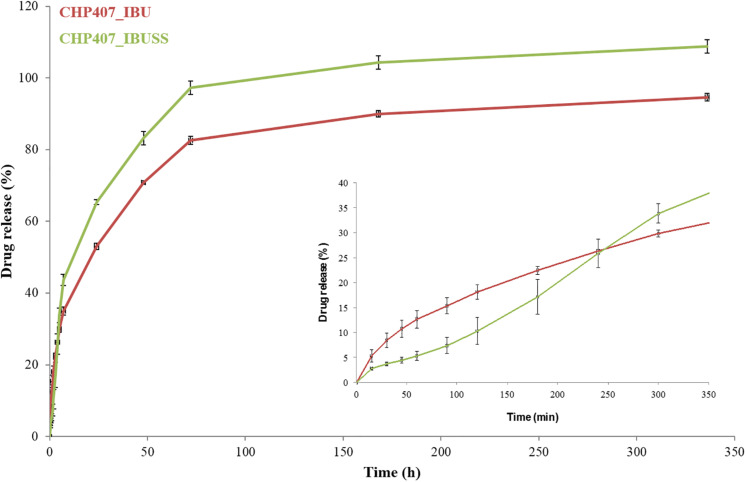
Drug release profile of IBU (red line) and IBUSS (green line) loaded at 1 mg/mL in 15% w/V concentrated CHP407 hydrogels. The insert magnifies drug release profiles within the first 7 h of incubation.

To study whether drug wettability could influence their release mechanism from micellar hydrogels, IBU and IBUSS, as hydrophobic and hydrophilic drug, respectively, were loaded in CHP407 gels at 1 mg/mL and their release kinetics was thoroughly investigated (Figure 7).

Irrespective of the nature of the encapsulated drugs, a sustained release over time was observed from CHP407-based hydrogels up to 14 days. In addition, starting from 7 h of incubation, IBUSS release (green line) was significantly higher than that of IBU (red line) at each analysed time point. However, considering the initial time steps up to 1 h, a delayed IBUSS release was registered followed by a sharp increase up to 7 h and finally by a sustained release which became complete on day 14. On the other hand, IBU-loaded hydrogels initially showed an increased payload release compared to IBUSS-loaded ones (8.5% versus 3.7% at 30 min of incubation) followed by a sustained release up to 14 days (approximately 95% released IBU). Thus, three and two different curve slopes could be identified in IBUSS and IBU profiles, respectively, which suggest the occurrence of different drug release mechanisms, and thus different release kinetics, as a consequence of drug wettability. In particular, 5 h incubation time marked a significant change in drug release kinetics as starting from this time point released IBUSS turned out to be always higher than IBU.

Lastly, to deeply investigate the mechanism underpinning the release of IBU or IBUSS within the first hours of incubation, the Korsmeyer–Peppas model was applied to drug-loaded CHP407 hydrogels (Dash et al., 2010). Specifically, the releasing exponent *n* was estimated for both CHP407_IBU and CHP407_IBUSS systems and they were measured to be 0.63 ± 0.007 and 0.89 ± 0.003, respectively. These results suggest the occurrence of two different release mechanisms of IBU and IBUSS from CHP407 gels, according to Dash et al. (2010).

## Discussion

Thermosensitive hydrogels are gaining increasing interest as drug delivery systems due to their capability to load high amounts of drugs. Moreover, the high water content of hydrogels perfectly mimics the extracellular matrix structure, thus reducing the probability of an adverse immune system response. However, the strong hydrophilic nature of these systems significantly limits the amount of hydrophobic drugs that can be encapsulated (Hoare and Kohane, 2008). To overcome this drawback, the introduction of hydrophobic blocks in the polymeric component can offer a higher number of binding sites to hydrophobic drugs, thus increasing the loading yield (Zha et al., 2002). However, this strategy generally requires a further functionalisation step. A promising alternative could be represented by micellar hydrogels, as their polymeric chains are composed by an alternation of hydrophobic and hydrophilic segments that upon temperature increase can arrange into organised structures with a hydrophobic core and a hydrophilic shell enhancing the interactions with drugs of different wettability.

In this work, the influence of drug nature on the temperature-driven micelle organisation and nucleation process has been thoroughly investigated at both the nano- and macro-scale. To this aim, an amphiphilic P407-based poly(ether urethane) was first synthesised and the success of its synthesis was assessed by ATR-FTIR spectroscopy. Indeed, the appearance of vibrational bands at 1,720 cm^–1^, 3,350 cm^–1^, and 1,540 cm^–1^ can be ascribed to the newly formed urethane bonds between isocyanate groups and hydroxyl terminal groups of the macrodiol and the chain extender in the first and second step of the synthesis, respectively. Furthermore, the absence of the peak attributed to unreacted isocyanates (i.e., 2,200 cm^–1^) suggested that the reaction was complete and successfully carried out as reported by Boffito et al. (2016). In addition, the band at 1,630 cm^–1^ suggested the presence of a small amount of urea bonds as a consequence of side-reactions occurring during PEU synthesis in accordance with the work recently published by Laurano et al. (2020). However, these by-products did not affect the successful synthesis of a poly(ether urethane) characterised by a high molecular weight and a narrow molecular weight distribution as assessed through SEC analyses.

The effects of drug wettability on polymer chain arrangement and micelle organisation were first investigated at the nanoscale by analysing two characteristic micelle parameters, the CMT and the average hydrodynamic micelle diameter. The former was estimated using a fluorescent dye (Alexandridis et al., 1994), and the latter by adapting to micelle characterisation a conventional technique (i.e., DLS analyses) usually applied to solid spherical systems (e.g., nanoparticles) (Hoo et al., 2008; Lim et al., 2013). Indeed, the presence of the amphiphilic P407 building block in polyurethane backbone is responsible for chain organisation into spherical micellar structures upon system heating according to results published on similar PEUs differing for their chain extender (Boffito et al., 2016, 2019a; Laurano et al., 2020). Drug encapsulation into CHP407 solutions affected the average hydrodynamic diameter of the polymeric structures formed at 25, 30, and 37°C, with significant differences that can be correlated to molecule wettability. Specifically, the addition of the hydrophilic IBUSS did not alter the measured micelle hydrodynamic diameters irrespective of temperature and its concentration within the sample, thus suggesting that no interactions occurred between IBUSS and micelle nucleation. Indeed, as a consequence of its hydrophilicity, IBUSS molecules preferentially interact with water molecules and/or chain hydrophilic segments. Conversely, IBU loading in CHP407 systems led to the formation of micelles characterised by higher hydrodynamic diameters with respect to the control (i.e., not-loaded CHP407 solutions). The formation of bigger polymeric structures as a consequence of IBU addition within the samples supported the hypothesis of the progressive hydrophobic drug arrangement into the hydrophobic micelle core upon temperature increase. Additionally, drug wettability also affected the temperature at which micelles began to aggregate (i.e., the CMT). Indeed, CMT is dependent on both polymer concentration, according to Boffito et al. (2016) and Laurano et al. (2020), and hydrophobic/hydrophilic balance (Okudan and Altay, 2019). In this work, being the polymeric content the same in all the analysed formulations, the progressive decrease of the estimated CMT values could be ascribed to the loading of increasing amounts of IBU. Indeed, the addition of hydrophobic drug molecules resulted in an increased system hydrophobic content, which led to the formation of stronger hydrophobic interactions and thus to the beginning of micelle aggregation phenomena at lower temperatures with respect to the control. On the other hand, the addition of IBUSS raised the hydrophilic content of the formulations, leading to a slight delay in polymeric chain arrangement into organised micelles with respect to the control. Additionally, this phenomenon was further worsened by the interactions occurring between drug molecules and chain hydrophilic blocks, which slightly hindered micelle formation. Therefore, based on these considerations and on the expected further decrease in CMT values moving from not-gelling solutions toward hydrogel formulations, in this work a new method was applied to prepare drug-loaded gels. Indeed, differently from the protocol we recently published (Boffito et al., 2019b), this new approach maximised IBU encapsulation within the hydrophobic micellar core during polymer solubilisation followed by its arrangement into micelles.

**FIGURE 8:**
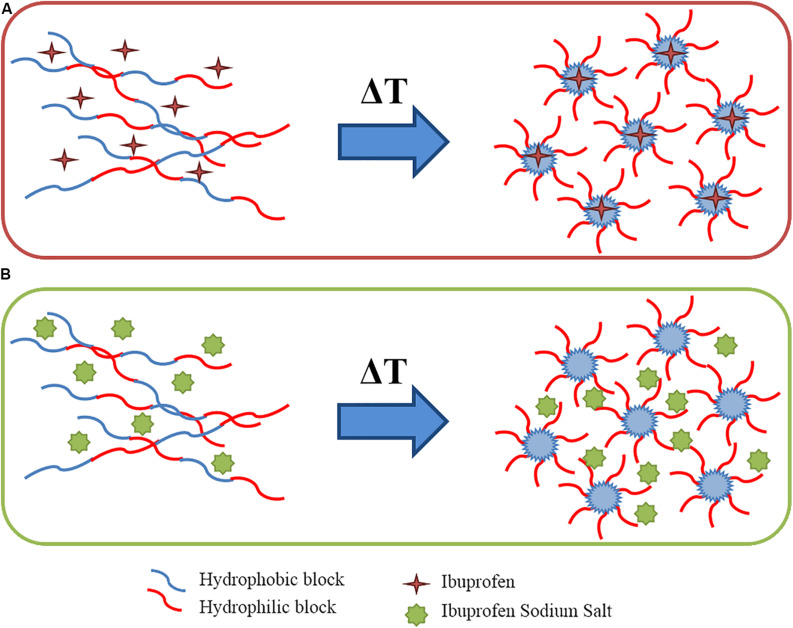
Schematic representation of wettability-driven drug arrangement during chain organisation into micelles upon temperature increase: hydrophobic drugs loaded inside the micelle core **(A)** and hydrophilic drug entrapped in the interstitial spaces among micelles **(B)**.

At the macro-scale the encapsulation of drugs in CHP407-based hydrogels, irrespective of their wettability, seemed to slightly interfere with the gelation temperatures as suggested by the Tube Inverting Test. This observation was further proved by temperature ramp tests, which revealed a decreased *T*_*onset*_ for both drug-loaded gels with respect to the CHP407 one. However, the addition of IBUSS only mildly altered the *T*_*onset*_ of the system, further supporting our observations at the nanoscale and definitely proving that hydrophilic drug molecules do not affect the organisation capability of polymeric chains. On the other hand, the addition of IBU drastically decreased the temperature at which micelles begin to aggregate. Indeed, IBU molecules, being encapsulated into the micelle core, as suggested by DLS measurements, led to the formation of bigger micelles reaching the critical micellar volume required to trigger the sol-to-gel transition at lower temperatures (Boffito et al., 2016). Additionally, the introduction of hydrophobic molecules into the system brought to increased hydrophobic interactions. This hypothesis was further confirmed by the measured strain at break, which was significantly lower for IBU-loaded gels with respect to IBUSS-loaded and virgin gels. Therefore, the addition of a hydrophobic drug moves the beginning of the gelation transition toward lower temperatures and induces the formation of less mechanically resistant gels. Concerning gel development kinetics, both drug-loaded hybrid systems were characterised by faster transitions with respect to the control. Moreover, this phenomenon was particularly evident in CHP407_IBUSS hydrogels. Indeed, although micelle aggregation began at lower temperatures for IBU-loaded gels with respect to IBUSS-loaded ones, at 25°C the hydrogel encapsulating the hydrophilic drug showed a marked lower crossover frequency. These observations could be explained considering the different drug arrangement within the network (Figure 8). Specifically, IBU molecules were encapsulated into the hydrophobic micelle core, leading to the formation of bigger micellar structures (Figure 8A), meanwhile IBUSS molecules were entrapped in the interstitial spaces among micelles (Figure 8B). IBU organisation into micelle core initially favoured the gelation process lowering the *T*_*onset*_, but then micelle nucleation and aggregation into the gel network turned out to be slightly hindered by their higher hydrodynamic diameters. On the other hand, hydrophilic drug arrangement with respect to the surrounding micelles did not alter *T*_*onset*_ value, but speeded up the sol-to-gel transition kinetics favouring micelle aggregation and packing into an organised network. However, at 37°C these differences between the analysed systems were levelled off as suggested by gelation time test results, ω_*G*__’__/G__”__*crossover*_ values, and temperature ramp test data. Indeed, although changes in the onset of gelation were observed, at temperatures higher than 30°C all the formulations tended to the same viscosity values. Thus, the here-developed micellar poly(ether urethane)-based hydrogels pave the way to the design of a drug delivery system able to encapsulate both hydrophilic and hydrophobic drugs without significantly altering the resultant hydrogel thermo-responsiveness.

In addition, the different wettability-driven drug arrangement into the gel network also affected their release kinetics from CHP407-based hydrogels. Specifically, the released amount of IBU turned out to be always lower than that of IBUSS from 7 h up to 14 days of incubation. Indeed, being encapsulated in the micelle core, drug molecules had a double diffusive barrier to pass through before reaching the external medium. However, this observation was not complied within the first 4 h of incubation as a consequence of the strong physical interactions occurring between IBUSS molecules and hydrophilic chain blocks, which led to a delayed release of the hydrophilic drug compared to the hydrophobic one (Hoare and Kohane, 2008). This different releasing mechanism of IBU and IBUSS from CHP407 gels was also supported by the estimated release exponent *n*. The Korsmeyer–Peppas model applied to CHP407_IBUSS gel release data within the first 7 h of incubation (being this model valid up to 60% release) evidenced an *n* value characteristic of case II transport. Indeed, IBUSS release was determined not only by diffusion and swelling/relaxation phenomena, but also by the occurrence of other processes influencing drug release, such as the electrostatic interactions occurring between IBUSS molecules and the polymeric chains (Dash et al., 2010; Boffito et al., 2019b). On the other hand, the Korsmeyer–Peppas model applied to CHP407_IBU systems gave an *n* value characteristic of non-Fickian transport (Dash et al., 2010; Boffito et al., 2019b). Indeed, the drug first diffused from the micelles within the hydrogel interstitial spaces and then from the gel in the surrounding medium. This result is in contrast with our recently published work (Boffito et al., 2019b), in which we characterised IBU release as a purely diffusive process. However, this contrasting data can be correlated to the different adopted protocol for drug loading. In our previous work, the addition of IBU to already solubilised CHP407 hydrogels led to only a partially effective loading of the drug within micelle core, being a fraction of the polymer chains already aggregated into micelles at the hydrogel preparation temperature (i.e., 4°C). Hence, a part of the added IBU probably remained in the hydrophobic micelle inlets and was released via diffusion mechanism. In contrast, in the present work the addition of IBU during polymer solubilisation allowed its direct incorporation into the micelles, which progressively assembled, leading to an anomalous releasing mechanism characteristic of drug delivery carriers with a double diffusive barrier (Boffito et al., 2019b). Thus, working on the loading protocol, hydrophobic drugs could be portioned among the different hydrophobic domains present within the gels, i.e., micelle core and inlets, with a consequent fine modulation of the release mechanism. CHP407 chain organisation into micelles upon temperature increase could thus be successfully exploited to load high amounts of hydrophobic molecules within the gels and avoid their burst release. Other parameters potentially affecting the release profile and mechanism are hydrogel composition, the initial cargo concentration within the gels, and the releasing volume surrounding them, which all together govern gel residence time and the forces involved in the progressive delivery of the payload from the systems to the surrounding watery environment.

This study has thus reported for the first time a thorough investigation, at both the nano- and macro-scale, of the interactions occurring between amphiphilic polymers and drugs with different wettability, which in turn strongly influence polymer chain arrangement in aqueous environment as well as cargo localisation within the formed micelle network and its release. All these aspects finally merge in the definition of a new hydrogel platform with the huge potential of loading high concentrations of drugs with different wettability and being *ad hoc* customised to deliver selected payloads with a well-defined time schedule.

## Conclusion

In this work, an amphiphilic poly(ether urethane)-based hydrogel was successfully developed, providing a step forward in the design of versatile drug delivery systems. According to the main goal of this investigation, IBU and IBUSS were selected as model drugs showing different wettability. In addition, they are widely employed in clinics as analgesic, anti-inflammatory, and antipyretic agents. Their loading within a vehicle phase opens the way to the possibility to locally release these drugs in target area, with the additional advantage of avoiding undesired side effects. The here-developed IBU- and IBUSS-loaded systems could thus find widespread application in the biomedical field in the treatment of many pathological states such as chronic wounds and musculoskeletal and rheumatic disorders. Our investigations evidenced that the characteristic PEU chain organisation into micelles in response to temperature increase could be exploited to encapsulate drugs with different wettability with no detrimental alterations of the thermo-sensitivity (i.e., all the resulting formulations retained the capability to gel in physiological conditions). In addition, the encapsulation of hydrophobic molecules within the micelles ensured prolonged drug release over time, thus avoiding their common burst release from hydrophilic networks. Furthermore, the different drug molecule arrangement within the gel network, as a consequence of their hydrophilic/hydrophobic nature and loading protocol, resulted in different drug releasing mechanism and timing. Thus, the here-developed platform could also open the possibility to design smart thermosensitive hydrogels able to simultaneously encapsulate and release high amounts of different therapeutic agents, thus eventually increasing the therapeutic efficacy through their co-current delivery into a target tissue/organ.

## Data Availability Statement

The datasets generated for this study are available on request to the corresponding author.

## Author Contributions

RL designed the experiments, analysed the data, and wrote the manuscript. MB designed the experiments, revised the manuscript, and supervised the whole work. Both authors contributed to the article and approved the submitted version.

## Conflict of Interest

The authors declare that the research was conducted in the absence of any commercial or financial relationships that could be construed as a potential conflict of interest.

## Footnotes

1 www.graphpad.com
