# Supplementary material for: Thermosensitive Micellar Hydrogels as Vehicles to Deliver Drugs With Different Wettability
Source: Front Bioeng Biotechnol. 2020 Jul 17;8:708. doi: 10.3389/fbioe.2020.00708 (PMC7379125; doi:10.3389/fbioe.2020.00708)
Supplement: Supplementary file 1 [file Table_1.pdf]

Supplementary Material

1 Poly(ether urethane) synthesis

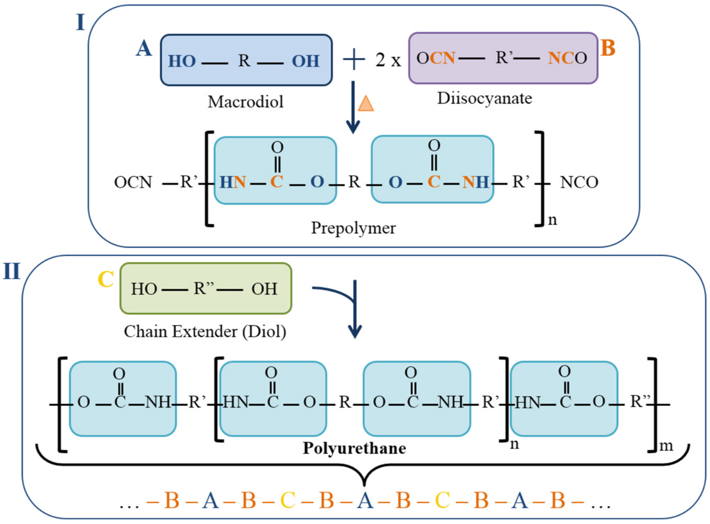

Supplementary Figure 1. Schematic representation of poly(ether urethane) synthesis carried out according to a two-step procedure.

2 Drug-loaded hydrogel preparation

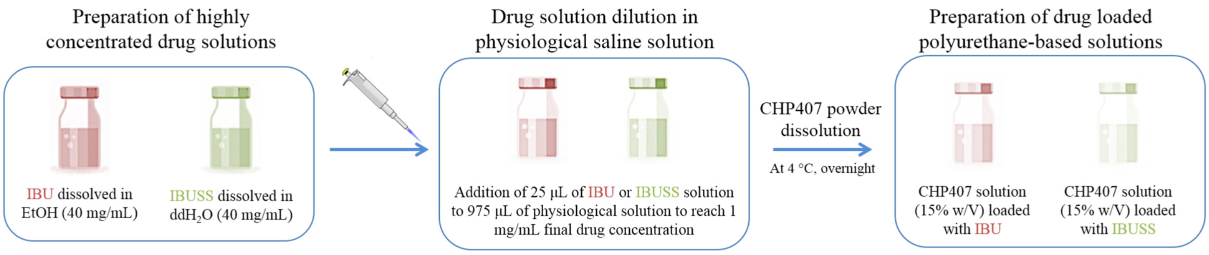

Supplementary Figure 2. Schematic representation of drug-loaded hydrogel preparation: drug dissolution at high concentration, drug dilution in physiological saline solution to reach the final desired concentration and polymer solubilisation in drug-containing physiological saline solution.

3 Poly(ether urethane) chemical characterization

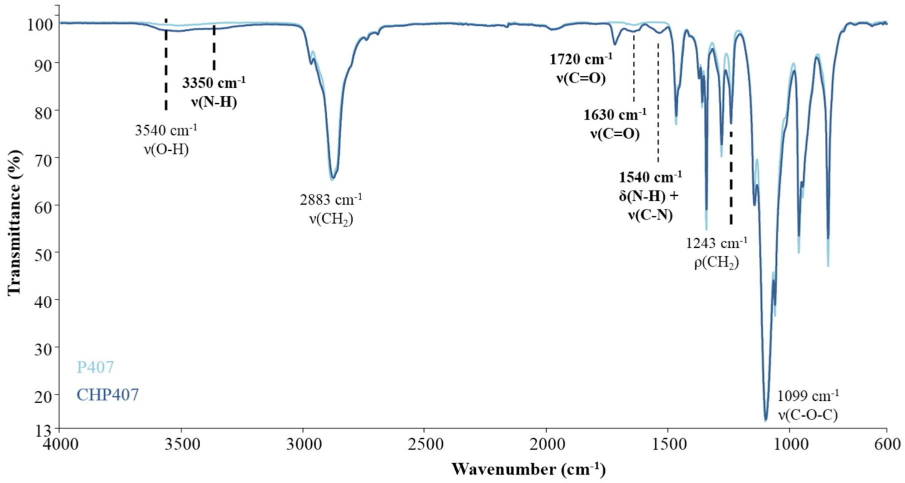

Supplementary Figure 3. ATR-FTIR spectra of P407 (light blue) and CHP407 (dark blue). New absorption bands highlighting the success of the synthesis are reported in bold.

4 Dynamic Light Scattering

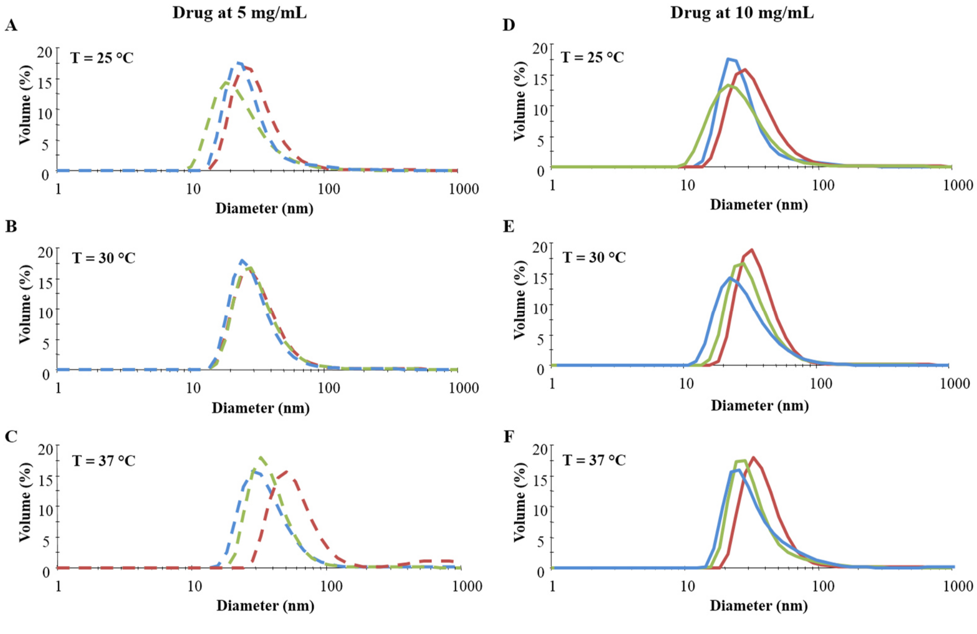

Supplementary Figure 4. Distribution patterns (by volume) of micelle hydrodynamic diameter measured in CHP407 solutions at 0.5% w/V concentration not loaded- (blue line) or loaded with 5 and 10 mg/mL of IBU (red line) or IBUSS (green line). Analyses were performed at 25 °C **(A, D)**, 30 °C **(B, E)** and 37 °C **(C, F)** *(n = 3)*.

5 Critical Micellar Temperature

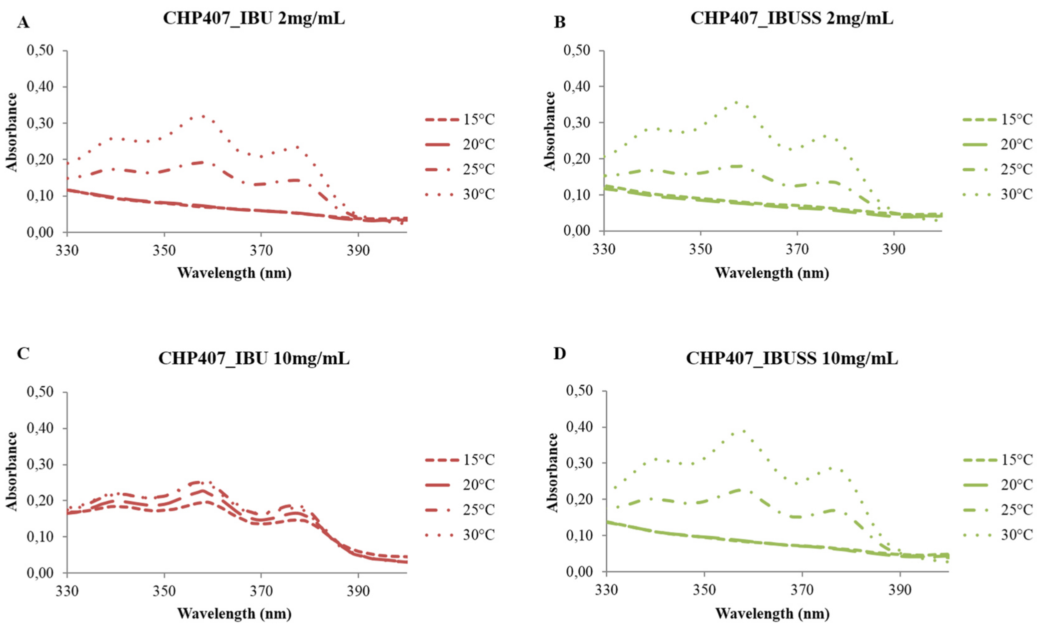

Supplementary Figure 5. UV/Vis spectra of CHP407 solutions at 0.5% w/V concentration loaded with IBU and IBUSS (red and green line, respectively) at 2 mg/mL **(A, B)** and 10 mg/mL **(C, D)**.

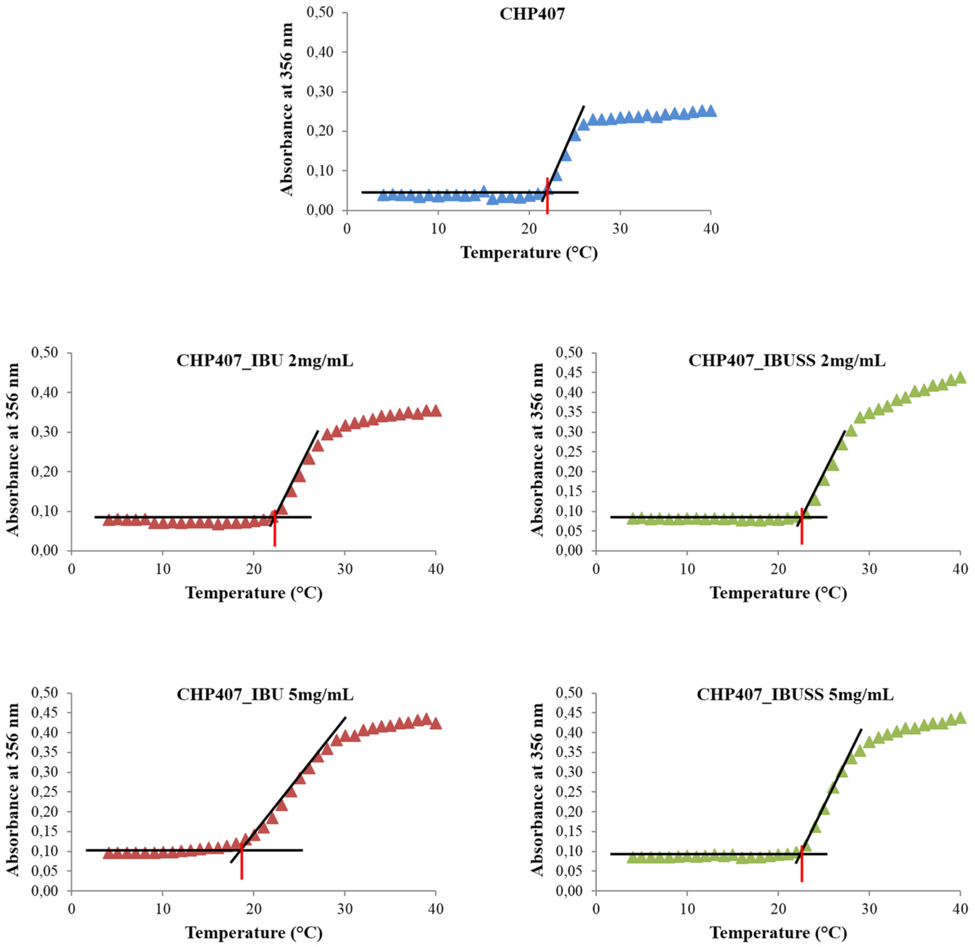

Supplementary Figure 6. Sigmoidal curves obtained by plotting the measured absorance values at 356 nm as a function of temperature for CHP407 solution (blue, 0.5% w/V polymer concentration) and CHP407_IBU (red) and CHP407_IBUSS (green) solutions loaded with drugs at 2 mg/mL and 5 mg/mL concentration.

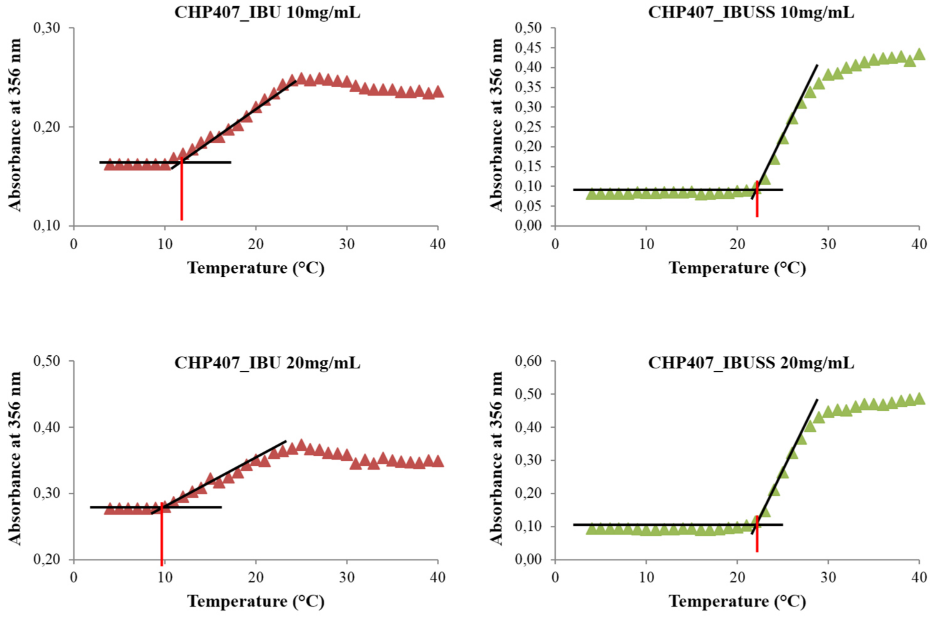

Supplementary Figure 7. Sigmoidal curves obtained by plotting the measured absorbance values at 356 nm as a function of temperature for CHP407_IBU (red) and CHP407_IBUSS (green) solutions loaded with drugs at 10 mg/mL and 20 mg/mL concentration.

6 Strain Sweep Test

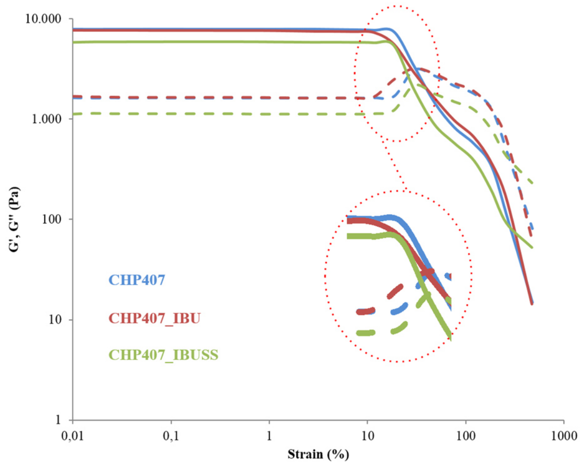

Supplementary Figure 8. Storage (G' – continous line) and Loss (G" – dashed line) moduli measured at 37 °C as a function of applied deformation in the range 0.01 – 500% for CHP407 (light blue), CHP407_IBU (red) and CHP407_IBUSS (green) systems.
